# Application of a three-session-procedure based on experiential learning in a tooth brushing course for Chinese dental students

**DOI:** 10.1186/s12909-019-1471-8

**Published:** 2019-02-04

**Authors:** Rui Zhang, Bin Zhang, Mingming Li, Jinfeng He, Tao Hu, Ran Cheng

**Affiliations:** State Key Laboratory of Oral Diseases & National Clinical Research Center for Oral Diseases & Department of Preventive Dentistry, West China Hospital of Stomatology, Sichuan University, Chengdu, Sichuan China

**Keywords:** Preventive dentistry, Modified bass technique, Electric toothbrush, Oral care, Dental students

## Abstract

**Background:**

Chinese dentists are obliged to provide reliable guidance to patients about tooth brushing. However, attitudes and behaviours of Chinese dental students regarding oral health have been insufficient. Traditionally, dental students were taught how to brush, but not how to evaluate tooth brushing. Here, we applied a three-session-procedure (TSP) based on experiential learning (EL) in a tooth brushing course for Chinese dental students. The aim was to improve dental students’ knowledge, practice of tooth brushing and self-evaluation, which may help cultivate their ability to conduct evaluation for friends, relatives and future patients.

**Methods:**

A quasi-experimental study design with a pre-test and post-test group was applied. A total of 176 students were enrolled in the TSP, which included a 1.5-h lecture course, a 3-h practice course for evaluation and comparison of manual and electric tooth brushing, and an after-class experience report. A survey including a knowledge test and a questionnaire on tooth brushing habits and opinions about the TSP was conducted 2 weeks later. The data about tooth brushing and the survey were collected and analysed.

**Results:**

Manual and electric tooth brush demonstrated almost equal overall efficiency in reducing plaque. However, for some students, either manual or electric tooth brushing was more suitable. Thus, it is advisable to estimate the exact differences in the efficiency of tooth brushing methods for each individual. The survey showed that tooth brushing by dental students significantly improved after TSP. The students could make self-evaluation and proper recommendations to family members and friends. The TSP was helpful in interpreting and evaluating manual and electric tooth brushing methods.

**Conclusions:**

A TSP course improved dental students’ knowledge, practice of tooth brushing and self-evaluation. In conclusion, a TSP based on EL is an effective and well-organized method of education on tooth brushing for Chinese dental students.

## Background

Microbial dental plaque harbour opportunistic pathogens that are the major aetiological agent in oral diseases, including caries and periodontal diseases [1]. The toothbrush, which dates to 3500–3000 B.C., is the most widely accepted and adopted tooth cleaning tool to achieve plaque control [2]. The 4th National Oral Health Survey in China demonstrated that poor oral hygiene behaviour, especially improper tooth brushing, is an important risk factor for dental caries [3, 4]. The Chinese oral public health service lacks specialists in oral health care and related oral health education for the populace [5]. It suggested that oral health education for tooth brushing should be focused on.

In China, dentists (including general dentists and specialists) are obliged to provide instruction on personal oral care to the patients. The guidance should cover the following: 1) manual or electric toothbrush selection, 2) tooth brushing techniques and 3) the effectiveness of tooth brushing. Moreover, Preventive Dentistry specialists should provide education and training in oral health promotion to a wide range of people, including in schools and communities. Oral health education from dentists or specialists is a way for professionals to affect people’s attitudes.

However, oral health attitudes and behaviours among Chinese dental students were insufficient. Chinese students had good knowledge about basic oral health measures; however their attitudes and practices towards oral health were relatively poor [6]. Dental students’ oral health education, attitudes and behaviours require strengthening [7]. Traditionally, education for undergraduate dental students included a lecture course and a practice course on the “modified Bass technique”, which is one of the most commonly recommended manual tooth brushing methods in China [8]. However, this traditional approach taught how to brush, but did not emphasize how to evaluate tooth brushing and thus was insufficient for dentists. Moreover, the modified Bass technique is comparably difficult to master, especially by children and disabled people [9, 10]. With the increasing acceptance of electric toothbrushes, more methods should be presented as candidate choices. Dental students should learn to give personalized advice about tooth brushing to their future patients. Thus, experience with and evaluation of different tooth brushing methods are essential for dental students.

Experiential learning (EL) is the process of learning through experience and is more precisely defined as “learning through reflection on doing” [11]. In the 1980s, David Kolb described a four-stage EL cycle, based on a combination of experience and subsequent reflection, that results in “real” learning [11, 12]. The EL cycle—experiencing, reviewing, concluding and planning—is suitable for dental students to learn, to summarize and to apply knowledge to future patients. It is therefore hypothesized that EL is applicable in tooth brushing education for Chinese dental students. We designed and analysed a three-session-procedure (TSP) based on EL composed of a lecture course (to learn theoretical knowledge), an experiencing course (to experience and review) and a report (to summarize). The 4th stage of EL, active experimentation was not evaluated in this study, but the students were encouraged to complete the fourth stage of EL, planning, by applying their learning in interactions with family members, friends, and future patients. The aim was to use TSP to improve dental students’ knowledge, practice of tooth brushing and self-evaluation. It was expected that the course will cultivate their ability to evaluate for friends, relatives and future patients.

## Methods

### Design

**Fig. 1:**
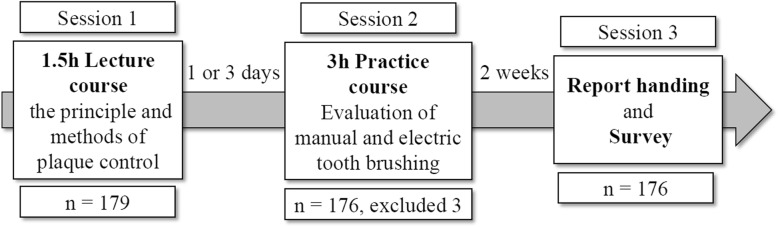
The flow chart of TSP

Randomization of individuals to treatment and control groups was not permitted because of ethical constraints. The research was conducted as a quasi-experimental study with a pre-test and post-test group. Based on EL, the three sessions of the TSP were arranged for the study as presented in Fig. 1.

### Participants

The inclusion criteria were: being a third-year dental student in West China Hospital of Stomatology, Sichuan University, and consent to participate in the program. The exclusion criteria included the following: 1. students whose Plaque Control Record was not appropriately written; 2. students who could not brush their teeth due to oral problems; and 3. students who were allergic to the Chrom-o-red Solution used during the TSP. In all, two students were excluded for inappropriate written records and one student was excluded due to a mucosal ulcer (unable to brush his or her teeth). According to data from the previous year, the plaque reduction rate by manual tooth brushing was approximately 70%. The desired sample size (*n* = 160) was calculated using the formula *n* = 2 × (U_α_ + U_β/2_) ^2^ × P(1-P)/δ^2^. A total of 176 third-year undergraduate dental students were eventually enrolled in the study (71 male, 105 female; aged 22.2 ± 0.3 years).

### Intervention

The TSP included three sessions. In the first session, students were divided into two groups and each group received a 1.5-h lecture course from the same instructor. The aetiology of periodontal disease, dental plaque and plaque visualization were introduced in 45 min. Subsequently, the principles and methods of plaque control were explained, e.g., tooth brushing (tooth brush, toothpaste, and brushing techniques), interproximal cleaning, rinsing and tongue cleaning. Finally, the question “which kind of toothbrush is suitable for you?” was raised.

The second session was executed within 3 days of the first session. The students were divided into four groups and each group had a 3-h practice course, all with the same instructor and teaching assistants. In the first 45 min of this session, the “modified Bass technique” [8], a modification of the Bass method combined with Roll method, was recommended as a manual tooth brushing method. Electric toothbrushes and the techniques recommended according to the manufacturer’s instruction (Philips Sonicare for teens HX6275, Koninklijke Philips N.V., Amsterdam, Netherlands) were also shown to the students. The Plaque Control Record [13, 14], which records the presence of dental plaque on four (buccal, lingual, mesial and distal) tooth surfaces, was introduced, as well. In the second and third 45-min segments of the class, students worked in pairs as a practising-detecting unit using Chrom-o-red Solution (Germiphene Corporation, Brantford, ON, Canada) to visualize dental plaque [15]. The student was required to stain dental plaque by rinsing his or her mouth with diluted Chrom-o-red Solution (1:20) for 1 min, followed by water. The detector recorded the presence of the plaque on individual tooth surfaces on a Plaque Control Record. The practiser randomly selected one side of the mouth for manual tooth brushing and the other side for electric tooth brushing. A total of 60 students had the left side of their mouths brushed with a manual toothbrush and the right side with an electric toothbrush, while 116 students had the left side brushed with an electric toothbrush and the right side with a manual toothbrush. According to the recommended brushing techniques, manual tooth brushing for half dentition took approximately 1~2 min, while electric tooth brushing lasted approximately 1 min. After tooth brushing, the detector recorded the residual plaque on another Plaque Control Record. Then, the students switched roles. The procedure was repeated until all the students had an opportunity to practise and detect. In the fourth 45-min segment, all students collected photos and analysed the data on the Plaque Control Record for their final report.

The third session of the TSP consisted of preparing for a report on the tooth brushing experience. Furthermore, the students were encouraged to extend the experience and make plans to instruct family members and friends after class. The reports were required to be submitted within two weeks.

### Instruments

We surveyed students using a survey instrument 2 weeks after the conclusion of the TSP. To ensure validity and reliability, the survey was analysed in single choices and was administered in class with adequate explanation. The first part included a 7-item knowledge test on the modified Bass technique. The second part contained 15 items on tooth brushing habits and self-estimation [according to a 5-point Likert type scale, from 1 (very bad) to 5 (very good)] before and after TSP. Different kinds of manual brushing methods, including the rolling method, Fones method and modified Bass method were surveyed. The recommended method for an electric toothbrush is usually consistent with its brand, only one method is recommended for the Philips electric toothbrush. The difference between various brands was not surveyed for these students. The last part of the survey solicited students’ opinions on the TSP, using a Likert type scale [1 (strongly disagree) to 5 (strongly agree)] to respond to 4 items pertaining to the course.

### Ethical consideration

This study was approved by the Institutional Review Board of the Ethics Committee of West China Hospital of Stomatology, Sichuan University (WCHSIRB-D-2018-092). Written informed consent was obtained from all participating students.

### Data collection and analysis

The content validity [16] of the questionnaire was assessed by 6 experts (1 professor, 1 associate professor and 4 lecturers). The reliability (Cronbach’s alpha coefficient) were analyzed by using SPSS 16.0 (IBM Corp. New York, NY, USA). Descriptive statistics were calculated and were presented as percentages, means and standard deviation (SD). Statistical analysis was performed using SPSS 16.0. To compare statistically significant differences, the Wilcoxon signed-rank test, Chi-square test and Fisher’s exact test were used. *P* < 0.05 was considered to indicate statistically significant differences.

## Results

**Fig. 2:**
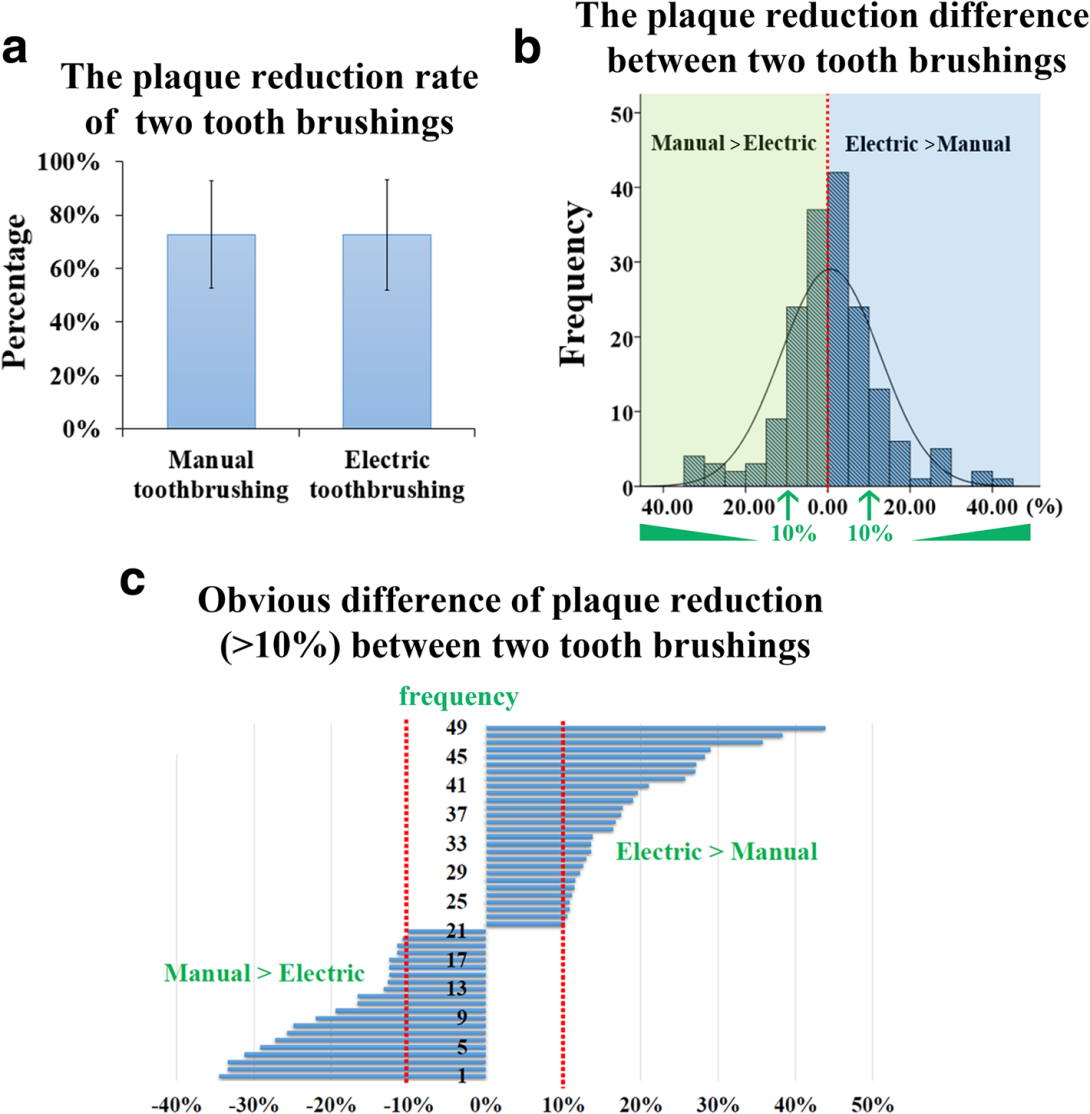
Comparison of manual and electric toothbrushes. **a** The reduction rate of plaque after manual or electric tooth brushing. There was no significant difference between the two (*P* = 0.679, Wilcoxon signed-rank test). **b** By comparing the two techniques, we subtracted the efficiency of manual and electric toothbrushes for each student. The differences in the frequency of toothbrush efficiency are also depicted. The green panel shows the efficiency and frequency of students whose manual toothbrushing overwhelmed electric tooth brushing (*n* = 82). The blue panel shows the students whose electric toothbrushing overwhelmed manual tooth brushing (*n* = 90). Only 4 students exhibited equal efficiency. **c** 49 of 176 students (27.27%) had an apparent difference (more than 10% of plaque removal) between manual and electric tooth brushing

A total of 176 third year undergraduate dental students participated in the TSP. Before tooth brushing, dental plaques were detected on 64.76% ± 25.21% surfaces of the right side (1, 4 regions) and 62.29% ± 25.88% on the left side (2, 3 regions). Comparing the right and left side using a Z-test found no significant difference (*P* > 0.05). The results showed that approximately 60% of the plaque was removed after correct tooth brushing. Manual and electric tooth brushing showed equal efficiency in reducing plaque (Fig. 2a). However, it was also suggested that a particular tooth brushing method was more suitable for the 27.27% (48/176) of students who exhibited apparent differences (more than 10% of plaque removal) between manual and electric tooth brushing (Fig. 2b, c). For these students, further evaluation of different tooth brushing methods should be conducted to determine an appropriate recommendation.

A content validity index (CVI) was calculated for questionnaire items. The item-level CVIs were higher than 0.83. The scale-level CVIs were 0.95. Reliability Analysis of the items about self-estimation and opinions showed that Cronbach’s Alpha = 0.71. For the subscales, Cronbach’s alpha was 0.71 and 0.81 for self-estimation items and options items. A Cronbach’s alpha greater than 0.70 was considered acceptable [17, 18]. As noted above, the first part of the questionnaire included an evaluation of manual tooth brushing using the modified Bass method. Questions 1–7 tested students’ knowledge of the details of brushing methods. As shown in Table 1, the average accuracy rate observed was 93.51%. Brushing effects when vibrating or rolling obtained the least accuracy (88.64%), which was because the answers should have been summarized and not refer to the text book. In all, most students had adequately learned the technique.

**Table 1:** The accuracy of test responses on the modified Bass method (*n* = 176)

Item	Content	n (%)
Question 1	The direction of the bristle	
	Accurate	166 (94.32)
	Wrong	10 (5.68)
Question 2	The angle of the bristle	
	Accurate	175 (99.43)
	Wrong	1 (0.57)
Question 3	The extent of brushing	
	Accurate	166 (94.32)
	Wrong	10 (5.68)
Question 4	The position of the brush head	
	Accurate	159 (90.34)
	Wrong	17 (9.66)
Question 5	Brushing time	
	Accurate	174 (98.86)
	Wrong	2 (1.14)
Question 6	Effect of brushing when vibrating	
	Accurate	156 (88.64)
	Wrong	20 (11.36)
Question 7	Effect of brushing when rolling	
	Accurate	156 (88.64)
	Wrong	20 (11.36)
Average	Accurate	164.58 (93.51)
	Wrong	11.42 (6.49)

**Fig. 3:**
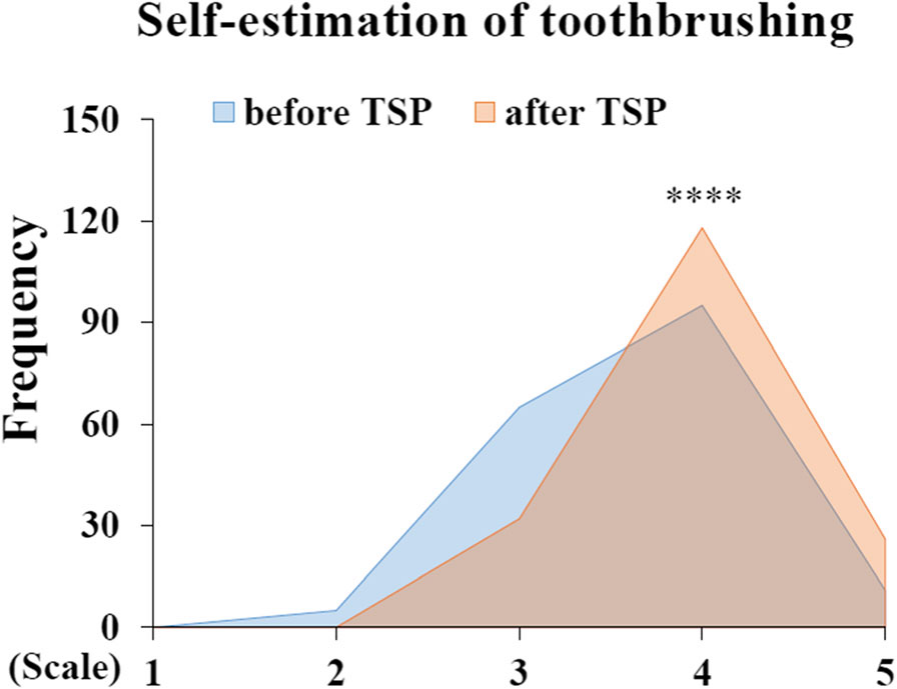
Self-evaluation of tooth brushing before and after TSP. The score of tooth brushing was elevated after TSP. Self-evaluation had also shown improvement. [****, Wilcoxon signed-rank test, *P* = 0.000; Scale: 1 (strongly disagree) to 5 (strongly agree)]

Furthermore, a survey on self-evaluation was conducted. Table 2 shows that the number of students who intended to use or were using electric toothbrushes increased from 49.43 to 74.43% (*P* = 0.000). Coverage of the modified Bass method increased from 69.32 to 93.18% after the TSP (*P* = 0.000). Nevertheless, some improper tooth brushing techniques, including the horizontal method, were also observed at the beginning of the lecture. The brushing time or frequency demonstrated by 1.14% of students was found to be inappropriate. Improper habits were addressed by showing “Do’s and Don’ts” and tooth brushing videos during the lecture session. Correct brushing action was demonstrated by the teacher or teaching assistants in the practice component of the course. After TSP, improper brushing methods, brushing time or frequency decreased to 0. The students rated their own tooth brushing methods before and after the class using a Likert-type scale from 1 (very bad) to 5 (very good). Their self-evaluations showed an increase in the score from 3.63 ± 0.64 to 3.97 ± 0.57, *P* = 0.000, suggesting that the students’ tooth brushing behaviour had improved (Fig. 3).

**Table 2:** Improvement of tooth brushing behaviour before and after TSP (n = 176)

Item	Before TSP	After TSP	*P*
	n (%)	n (%)	
Use or intent to use electric toothbrush	87 (49.43)	131 (74.43)	0.000^a^
Use modified Bass method	122 (69.32)	164 (93.18)	0.000^a^
Improper tooth brushing technique	3 (1.70)	0 (0)	0.248^b^
Improper tooth brushing time (< 2 min)	2 (1.14)	0 (0)	0.499^b^
Improper brushing frequency (< 2 times/day)	2 (1.14)	0 (0)	0.499^b^

^a^Chi-square test; ^b^Fisher’s exact test

The students were also able to offer better advice or new plans with family members and friends. Table 3 presents possible advice for these groups. While recommendation of electric toothbrushes increased from 80.11 to 86.93%, this was not a statistically significant difference (*P* = 0.085). Recommendation of the modified Bass method significantly increased, from 81.81 to 94.89% (*P* = 0.000). Improper recommendations had been corrected as well.

**Table 3:** Recommendations about tooth brushing made to family or friends before and after TSP (*n* = 176)

Item	Before TSP	After TSP	*P*
	n (%)	n (%)	
Electric toothbrush	141 (80.11)	153 (86.93)	0.085^a^
Modified Bass method	144 (81.81)	173 (94.89)	0.000^a^
Improper brushing technique	3 (1.70)	0 (0)	0.248^b^

^a^Chi-square test; ^b^Fisher’s exact test

Lastly, the students’ opinions on the TSP itself were collected (Table 4). The TSP received a high score. Students reported that all sessions were remarkably helpful regarding interpreting and evaluating manual and electric tooth brushing methods.

**Table 4:** Opinions towards TSP

Item	Mean score	SD
The homework report was helpful	4.83	0.42
The lecture was helpful	4.89	0.37
The practice was helpful	4.54	0.82
TSP improved learning	4.88	0.42

## Discussion

### The course on tooth brushing was important for dental students

Appropriate tooth brushing techniques are very important to maintain oral hygiene. Thus, the course on tooth brushing was conducted as an integral part of Preventive Dentistry. Preventive Dentistry, a mandatory and specialized course for dental students, begins in the spring term of the third year undergraduate program at Sichuan University and is the first course about dentistry. The students have little knowledge of dentistry at this stage. Therefore, for dental students, the importance of the course on tooth brushing was not merely on technique. It essentially requires students to experience, compare and summarize. Moreover, it offers the first opportunity for prospective dentists to develop appropriate professional advice or plans for future patients.

The choice of a tooth brushing method should be a personal selection. Previous studies have suggested that the electric toothbrush showed greater plaque reduction compared to the manual toothbrush [19, 20]. Similar results were reported in young adult patients [21] and disabled school children [22]. Oscillating-rotating tooth brushing (another modified Bass method) reduced both plaque and gingivitis compared to an electric toothbrush in a long-term (12 week) study [23]. However, no significant difference was observed between manual and electric tooth brushing techniques in orally healthy young adults [24]. Our results are consistent with that study, also finding no significant difference between manual and electric tooth brushing techniques in the third year undergraduate dental students. Although majority of the students had no significant difference in plaque clearance, 27.27% (48/176) of students showed obvious differences in plaque clearance between manual and electric toothbrushes (Fig. 2b, c). Both manual and electric tooth brushing techniques are effective in plaque clearance. But for individual selection, personal evaluation and effectiveness should be taken into consideration.

The results further verified that both experience and evaluation are essential for successful education. The theory of EL is based on the concept that “learning is the process whereby knowledge is created through the transformation of experience” [11]. EL is known to be valuable in medical education [25]. Moreover, EL has been found to be more successful than traditional methods of learning in oral hygiene education and improvement in children [26, 27]. For experiencing, comparing, summarizing, and solving patients’ problem, Kolb’s experiential learning theory (ELT) was found to be applicable in our education system. The theory presents a cyclical model of learning comprising four stages. The first stage, concrete experience, is when the learner actively experiences an activity, as during a lab session or fieldwork. The second stage of ELT is reflective observation, when the learner consciously reflects back on the concrete experience. In the third stage, abstract conceptualization, new theories are conceptualized based on the learner’s reflection, or modifications are applied to the existing theory. The fourth stage, active experimentation, to test a model or theory or plan for an upcoming experience.

Our TSP was designed based on EL. First, the practice course of TSP was the first ELT stage, in which students experienced both manual and electric tooth brushing. By staining plaques before and after tooth brushing, students could clearly see retained dental plaque, indicating poorly cleaned teeth. Following the practice course, the students submitted reports based on their experiences, summarizing the effect and experience of both tooth brushing methods. The students then determined which toothbrush was appropriate for them based on their experience. In doing so, the purpose of the second stage of ELT was accomplished. Furthermore, by seeing the remaining dental plaque, students could improve their brushing techniques by, for instance, using a small head toothbrush with raised cleaning tip to access posterior parts of the mouth [28]. These personalized alterations will influence the goal of the third ELT stage. The fourth ELT stage, active experimentation, was informally encouraged when the students were prompted to make new plans for their family members and friends. They would also be able to begin developing their approaches and practices towards patients, which they may replicate when they become professional dentists.

A survey was conducted to evaluate the efficiency of the TSP. The findings of the present study indicated that most of the students efficiently learned the modified Bass method and incorporated the theory into the routine experience. The electric toothbrush was increasingly accepted after TSP. Moreover, improper tooth brushing methods were corrected. In all, the TSP was accepted by the dental students. Our results indicate the students improved their personal knowledge, practice and self-evaluation by using TSP. Their improved knowledge may have the potential to cultivate their abilities to conduct evaluation with friends, relatives and future patients.

### Limitations

Due to ethical concerns, it was not practical to use a control group. Instead, a quasi-experimental study was used. Brushing methods for electric brushes vary among different design and brands; this might be a topic for future studies. The fourth stage of ELT, in which the learner plans to test a model with an upcoming experience, was not assessed in the present study. We encouraged the students to complete the fourth stage by making plans for their family members and friends; however, the students were not asked to submit reports on this stage.

## Conclusions

The TSP improved dental students’ knowledge, practice of tooth brushing and self-evaluation. The fourth stage of ELT would be fulfilled when students make new plans with friends, relatives and future patients. In conclusion, the TSP based on EL is an effective and well-organized method of education on tooth brushing for dental students.

## Abbreviations

CVI: Content validity index
EL: Experiential Learning
ELT: Experiential Learning Theory
SD: Standard Deviation
TSP: Three-session-procedure
